# Favorable Virological Outcome, Characteristics of Injection Site Reactions, Decrease in Renal Function Biomarkers in Asian People with HIV Receiving Long-Acting Cabotegravir Plus Rilpivirine

**DOI:** 10.1089/aid.2023.0108

**Published:** 2024-04-11

**Authors:** Eisuke Adachi, Makoto Saito, Amato Otani, Michiko Koga, Hiroshi Yotsuyanagi

**Affiliations:** Department of Infectious Diseases and Applied Immunology, IMSUT Hospital of The Institute of Medical Science, The University of Tokyo, Tokyo, Japan.

**Keywords:** cabotegravir plus rilpivirine, long-acting drug, injection site reaction, HIV, antiretroviral therapy

## Abstract

Long-acting cabotegravir plus rilpivirine has revolutionized the concept of antiretroviral therapy, but as the causes of virological failure and satisfaction can depend on patient background, real-world data are needed. In this single-center study, we reviewed clinical records of people with HIV (PWH) who received injectable cabotegravir plus rilpivirine between June 2022 and January 2023. We assessed virological and safety outcomes, including injection site reactions (ISRs) and changes in serum creatinine and cystatin C. Seventy-four patients were included. There were no virological failures. Approximately 80% of individuals achieved HIV-RNA undetectable in all visits up to 14 months (median 13 months) after switching. Pain upon injection was significantly more common at the rilpivirine injection site, while delayed pain was significantly more common at the cabotegravir injection site. The serum creatinine (mean difference −0.12 mg/dL, *p* < .0001) and the cystatin C (mean difference −0.077 mg/dL, *p* < .0001) decreased significantly after switching, and in multivariable regression analysis, baseline characteristics did not affect the decrease in these renal function markers. Long-acting cabotegravir plus rilpivirine showed excellent antiviral efficacy and safety in PWH in Japan. ISRs were characterized differently at the cabotegravir and rilpivirine injection sites. Although cystatin C showed decrease after the regimen switch, further confirmation is needed whether cabotegravir plus rilpivirine can improve renal function.

## Introduction

A long-acting therapy of cabotegravir plus rilpivirine (CAB plus RPV LA) is now available for treatment with intramuscular administration every month or every 2 months.^1,2^ Since its approval in the United States and Europe, there has been controversy as to which people with HIV (PWH) would benefit from this regimen. Patient satisfaction was high, with over 90% preferring the injectable long-acting agent over the oral drug.^2,3^ However, to date, real-world data on this issue were scarce. The effectiveness of CAB plus RPV LA can vary from region to region, and differences in health care systems can affect patient satisfaction.

In Japan, CAB plus RPV LA became available in late June 2022. In our previous report, we showed that even patients who would have to go to the hospital more frequently after switching to CAB plus RPV LA, and even patients who would not eliminate their oral medications due to other concomitant oral medications, felt that they would benefit from switching to CAB plus RPV LA.^4^

Although CAB plus RPV LA has revolutionized ART by being an injectable drug, the impact of injection site reaction (ISR) on the lives of PWH has never been discussed fully, because no previous options included injectable drugs. ISR was a key endpoint in clinical trials,^2,3,5^ however, it is based on subjective symptoms and may have a different impact on PWH in the real world, where regimens can be more easily switched than in clinical trials, which are contract based. There are racial and cultural differences in ISR and pain perception.^6^ Clinical data on adverse reactions other than ISR are also scarce. Although dolutegravir (DTG) and bictegravir are known to increase serum creatinine by inhibiting tubular transport proteins, no clinical data have been reported for CAB.

The objective of this study is to evaluate the satisfaction and acceptability of CAB plus RPV LA in Japan by determining the virological efficacy, safety profile (e.g., renal function biomarkers), and ISR after switching to CAB plus RPV LA.

## Patients and Methods

### Population

In this single-center study, we reviewed routinely collected clinical records of PWH who received at least one dose of injectable CAB plus RPV LA at IMSUT hospital of the Institute of Medical Science, The University of Tokyo, between June 2022 and January 2023. Laboratory data through September 2023 were included in the analysis. Treatment was given in accordance with the Japanese drug package inserts: all patients had been on oral CAB plus RPV LA for at least 1 month and had maintained HIV-RNA <50 copies/mL for at least 6 months before the oral lead-in (OLI) initiation. We introduced CAB plus RPV LA if the patient is virologically suppressed, regardless of anti-hepatitis B surface antibody status or oral adherence. Patients of non-Asian ethnicity, patients with a history of participation in clinical trials, and patients who used these drugs in a way other than that described in the package insert were excluded from the analysis.

### Evaluation of virological outcomes

Virological outcomes were evaluated at the start of OLI, at the start of injectable CAB plus RPV LA, and at 1, 3, 5, 7, 9, 11, and 13 months after injectable CAB plus RPV initiation, that is, ∼14 months after the start of OLI. One who was dosed every month was analyzed in the same way. HIV-RNA was measured at BML, Inc. (Tokyo, Japan), using cobas^®^ 6800/8800 System (Roche diagnostics, Tokyo, Japan); HIV-RNA ≥20 copies/mL was measured quantitatively and HIV-RNA <20 copies/mL was evaluated for qualitative detection. “Undetectable HIV-RNA” means that the virus is qualitatively undetectable, which is synonymous with “Target not detected.”

### Evaluation of ISRs

The injection site pain was recorded with severity for each drug. The severer side was used for the analysis. If the pain was the same on both sides, only the pain that concerned the participant was counted. Pain was differentiated depending on the timing of appearance: pain upon injection referred to the acute pain felt at the time of injection and delayed pain is the pain that developed on or after the day following injection. Duration of the pain was counted in days; if there was no pain on the next day, it was counted as 0 days. Duration was reported as median and interquartile range (IQR), excluding those who did not complain of pain. Systemic symptoms such as pyrexia and fatigue were also investigated in the same way if they were associated with injections.

### Evaluation of renal function biomarkers

At each visit from the start of OLI until 7 months after injectable CAB plus RPV administration, that is, ∼8 months after the start of OLI, data on serum creatinine and cystatin C were collected from clinical records to assess safety. To evaluate renal function biomarkers in stable conditions, only test results conducted at regular HIV outpatient visits were included, and the results at the irregular visits for other complaints (e.g., acute infectious diseases) and at the follow-ups for complications were excluded from the analysis.

### Statistical analyses

We used the exact McNemar test for comparing the injection site pain between CAB and RPV. One-way analysis of variance was used to test for differences in ISR duration between the respective drug injection sites. Repeated measures analysis of variance (ANOVA) was used to assess whether serum creatinine and cystatin C changed statistically at different time points. We used the paired *t*-test to evaluate the difference between biomarkers before (baseline OLI initiation) and 3 months after switching to CAB plus RPV LA. Cumulative probability of patients whose HIV-RNA remained not detected after the start of injectable CAB plus RPV LA was evaluated using Kaplan–Meier estimates: only patients with undetectable HIV-RNA at baseline were included. Statistical analyses above were performed using Stata/MP 16.1 (StataCorp) or Prism 9 (GraphPad Software).

Multivariable regression analysis was performed with the difference between renal function biomarkers before (baseline OLI initiation) and 3 months after switching to CAB plus RPV LA as the dependent variables and age, body mass index (BMI), inflammation biomarker (i.e., CD4/CD8 ratio and C-reactive protein), smoking, heart diseases, malignancy and preswitching regimens (i.e., DTG-containing regimen, bictegravir/emtricitabine/tenofovir alafenamide (B/F/TAF) use, cobicistat-containing regimen, and other regimens), as the independent variables, using JMP Pro 17 (SAS Institute, Inc.). Analysis of covariance (ANCOVA) was also conducted using the same variables.^7^ Statistical significance was defined as two-sided *p* < .05.

## Results

### Population

Eighty-two PWH received CAB plus RPV LA during the period, and eight of whom were excluded: four were clinical trial participants and had been administered before the time period of this study; two were non-Asian ethnicity; one was on OLI only for 1 week due to an underlying condition that made oral administration difficult; and one switched to the previous regimen after the OLI because of fatigue. A total of 74 PWH were analyzed. One received CAB plus RPV LA every month and 73 received CAB plus RPV LA every 2 months. Baseline characteristics of the participants are shown in Table 1. Among the participants, only three were women, one of whom was a transgender woman. The majority of participants was Japanese and infected with HIV-1 subtype B, which is not different from the distribution among PWH in Japan. Participants with subtypes AG, AE, D, and G were all Japanese, and some of these participants were thought to be infected by non-Japanese. All patients had HIV-RNA <50 copies/mL at the start of OLI, but 2 had HIV-RNA ≥50 copies/mL at the start of CAB plus RPV LA injection.

**Table 1.** Baseline and Demographic Characteristics

Total,* n*	74
Age, years	47 [26–64]
Gender	
Male	71
Female^a^	3
Race	
Asians	73
Southeast Asians	1
CD4+ cell count, cell/μL	647 [200–1,210]
HIV-RNA	
<50 Copies/mL	72
≥50 Copies/mL	2
Body mass index, kg/m^2^	24.1 [17.7–36.8]
>30 kg/m^2^	4
HIV-1 subtype^b^	
B	68
AG	2
AE	1
D	1
G	1
ART regimen before switching	
DTG/3TC	28
B/F/TAF	33
ABC/3TC/DTG	2
F/TAF+DTG	2
D/C/F/TAF or DRV/COBI	2
Other regimens^c^	4
Hepatitis B status	
Positive for anti-HBs	51
Negative for anti-HBc	22

Data are *n* or median [range].

^a^ 1 for transgender woman.

^b^ No data for 1 individual.

^c^ 2 for rilpivirine/emtricitabine/tenofovir alafenamide and 2 for raltegravir + emtricitabine/tenofovir alafenamide.

ABC/3TC/DTG, abacavir/lamivudine/dolutegravir; anti-HBc, anti-hepatitis B core antibody; anti-HBs, anti-hepatitis B surface antibody; ART, antiretroviral therapy; B/F/TAF, bictegravir/emtricitabine/tenofovir alafenamide; D/C/F/TAF, darunavir/cobicistat/emtricitabine/tenofovir alafenamide; DTG/3TC, dolutegravir plus lamivudine; DRV/COBI, darunavir/cobicistat.

Three patients switched to other oral ART within 3 months after initiation because of ISR; the ISRs for all three cases were mild, equivalent to grade 1 in general clinical trials grading. There were no cases of switching from CAB plus RPV LA to other regimens due to drug-related adverse events other than ISR.

### Antiviral efficacy of cabotegravir plus rilpivirine

**FIG. 1.**
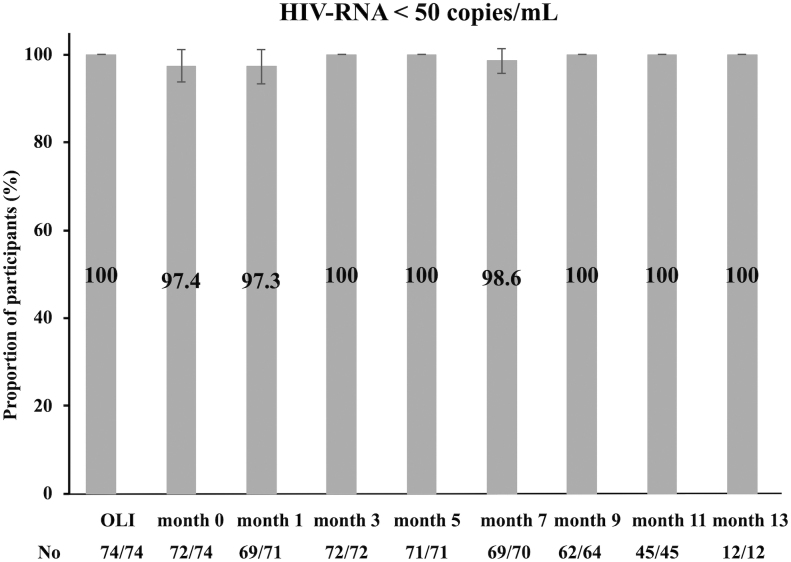
Proportion (95% confidence interval) of patients with HIV-RNA less than 50 copies/mL at each visit. “OLI,” the start of the oral lead-in. “month 0,” the start of cabotegravir plus rilpivirine injectable.

**FIG. 2.**
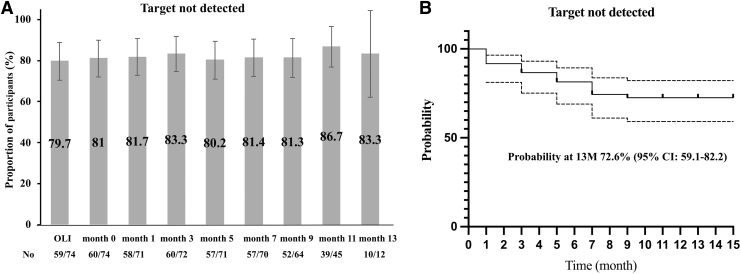
Proportion (95% confidence interval) of patients with target not detected at each visit **(A)** and the Kaplan–Meier estimates of the probability of maintenance of undetectable HIV-RNA among patients whose HIV-RNA was undetectable at the start of cabotegravir plus rilpivirine injectable **(B)**. “OLI,” the start of the oral lead-in. “month 0,” the start of cabotegravir plus rilpivirine injectable.

The median observation period for this study was 14 months (1 month for OLI and 13 months for CAB plus RPV injectable). More than 97% of individuals had maintained HIV-RNA <50 copies/mL in all visits up to 13 months after the administration of injectable CAB plus RPV, and no individuals had virological failure (Fig. 1). Figure 2A shows the percentage of HIV-RNA achieving less than detection sensitivity at each visit. Approximately 80% (60/74) of individuals were with undetectable HIV-RNA (i.e., “target not detected”) at the start of OLI or at the start of injectable CAB plus RPV LA, and this was well maintained after switching to CAB plus RPV injectable. Cumulative probability of individuals with target not detected was 86.7% (95% confidence interval [CI]: 75.1–93.0) at month 3 and 72.6% (95% CI: 59.1–82.2) at month 13, by the Kaplan–Meier estimates (Fig. 2B).

Figures 1 and 2 have missing data for the following reasons: in month 1, unmeasured HIV-RNA (three cases); in month 3, discontinuation of CAB plus RPV (two cases); in month 5, discontinuation of CAB plus RPV (three cases); and after month 7, discontinuation of CAB plus RPV (three cases) and absence from outpatient visits during the study period (X cases).

### Characteristics of ISRs at rilpivirine and cabotegravir injection sites

Eighty-six percent (64/74) of the patients reported any ISRs at the first administration, and 62.0% (44/71) after the second administration. At the time of the first injection, 74.3% (55/74) reported pain upon injection and 39.1% (29/74) had delayed pain, whereas, at the time of the second injection, 52.1% (37/71) had pain upon injection and 26.8% (19/71) had delayed pain. Table 2 shows that pain upon injection was significantly more common at the RPV injection site, while delayed pain was significantly more common at the CAB injection site for the first administration. The same significant difference was found for the second injection. The duration of pain was similar on the CAB side, the RPV side, and both sides, with a median of 2 days for pain upon injection and 3 days for delayed pain and was similar between the first and second injections. Pyrexia was observed in 2.1% (3/145) of individuals either at the first and second injections, and pyrexia and fatigue combined were observed in 4.8% (7/145) of patients.

**Table 2.** Injection Site Reaction After the First or Second Administration

	First dose (*n* = 74)	*p*	Second dose (*n* = 71)	*p*
Number of participants who reported ISR	66		44	
Pain upon injection				
CAB > RPV	2	.00040^a^	2	.013^a^
RPV > CAB	18		12	
Equal on both sides	35		23	
Duration (pain upon injection), days (IQR)				
Pain of any patterns	2.0 [0–3.0]		1.0 [0–3.0]	
CAB > RPV	3.0 [3–3]	.58^b^	1.5 [0.8–2.6]	.34^b^
RPV > CAB	2.5 [0.3–3.0]		0.5 [0–1.5]	
Equal on both sides	2.0 [0–3.0]		1.0 [0–3.0]	
Delayed pain^c^				
CAB > RPV	14	.013^a^	8	.039^a^
RPV > CAB	3		1	
Equal on both sides	12		10	
Duration (delayed pain^c^), days				
Pain of any patterns	3.0 [3.0–3.8]		3.0 [2.0–7.0]	
CAB > RPV	3.0 [3–4.5]	.86^b^	2.0 [2.0–3.0]	.083^b^
RPV > CAB	3.0 [3.0–3.0]		7.0 [7.0–7.0]	
Equal on both sides	3.0 [1.8–3.5]		5.0 [3.0–7.0]	
Nodule	4		4	
Pyrexia or fatigue	6		1	
Pyrexia	2		1	

Data are *n* or median.

^a^ Pain that developed on or after the day following injection: ISR, CAB, and RPV.

^b^ Exact McNemar test.

^c^ One-way analysis of variance.

CAB, cabotegravir; IQR, interquartile range; ISR, injection site reaction; RPV, rilpivirine.

### Changes in renal function biomarkers after switching

**FIG. 3.**
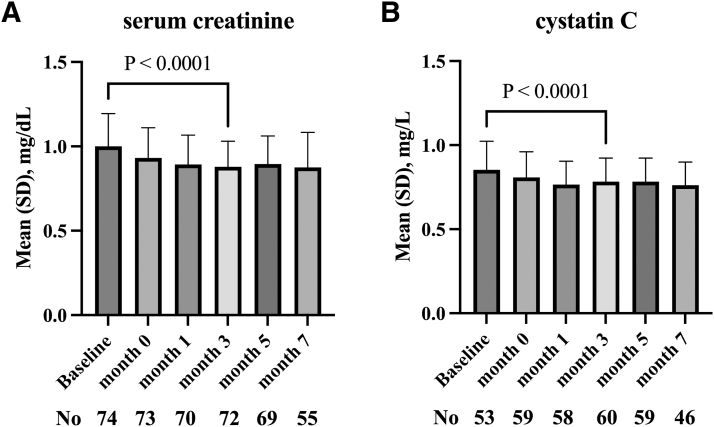
The changes in renal function biomarker after switching to cabotegravir plus rilpivirine. Serum creatinine **(A)** and cystatin C **(B)** are shown. The *p* values in the graphs are calculated by paired *t*-test comparing the values at baseline and at month 3. “Baseline,” at the start of oral-lead-in.

Any changes in renal function biomarkers after switching to CAB plus RPV LA were examined using repeated measures ANOVA. There were significant changes in both serum creatinine (*p* < .0001; Fig. 3A) and cystatin C (*p* < .0001; Fig. 3B). The serum creatinine and cystatin C decreased after the introduction of OLI and then leveled off rather than continuously decreasing over time. To perform a repeated measures ANOVA for this change, it was considered appropriate to perform the analysis of variance at month 7, when sufficient data are available, rather than comparing up to month 13, when fewer data are available.

Additionally, when the serum creatinine and cystatin C were compared specifically between baseline OLI initiation and month 3 by paired *t*-test, both serum creatinine (mean difference −0.12 mg/dL, *p* < .0001 [*n* = 72]) and cystatin C (mean difference −0.077 mg/dL, *p* < .0001 [*n* = 53]) were significantly decreased. Serum creatinine and cystatin C were significantly reduced regardless of the original ART regimens before switching. None of the participants' statuses of chronic inflammation, heart disease, malignancy, or smoking changed from the baseline.

To further investigate the impact of these baseline factors on the absolute change in renal function biomarkers, we evaluated the effect of age, BMI, inflammation biomarker (i.e., CD4/CD8 ratio and C-reactive protein), smoking, heart diseases, malignancy and preswitching regimens (i.e., DTG-containing regimen, B/F/TAF use, cobicistat-containing regimen, and other regimens) using multivariable regression analysis. None of the factors had a statistically significant effect on the changes in creatinine and cystatin C. Same conclusion was made by using an analysis of covariance (ANCOVA).^7^

## Discussion

The observation period was determined based on when virological failures occurred in clinical trials. Of the cases of confirmed virological failure by the primary endpoint of week 48, in the ATLAS trial, all three of the cases occurred by week 24,^8^ in the FLAIR trial, 3 of 4 cases developed virological failure by week 28,^9^ and in the ATLAS-2M trial 9 out of 10 cases had occurred by week 24.^1^ The median observation period of this study was able to detect 88% (15/17) of virological failures in the three clinical trials.^1,8,9^ The tendency to virological failure shortly after treatment initiation is a characteristic feature of CAB plus RPV LA. Therefore, our study had an observation period with sufficient margin to detect virological failures.

Pooled data from the phase 3 and phase 3b ATLAS, FLAIR, and ATLAS-2M trials suggested that risk of confirmed virological failure increased when two or more of the following baseline factors were present: proviral rilpivirine resistance-associated mutations, HIV-1 subtype A1/A6, or a BMI of 30 kg/m^2^ or more.^10^ Most of the patients on CAB plus RPV LA included in this report were infected with subtype B. In addition, among the three phase 3 trials, one case of virological failure occurred after 96 weeks of ATLAS-2M in a patient with subtype B infection.^2^ Some participants with HIV-1 subtype B failed virologically in the SOLAR study, a head-to-head trial comparing with B/F/TAF,^11^ and in the CARLOS study, which evaluated CAB plus RPV LA up to 6 months in a real-world setting.^12^ Our study showed that the risk of virological failure is small in real-world data from Japan, where HIV-1 subtype B is endemic. Participants in this study also had a median BMI of 24 kg/m^2^ and 4 cases with BMI ≥30 kg/m^2^, all of which were virologically suppressed.

In recent clinical trials, more sensitive virological markers other than conventional endpoints (e.g., HIV-RNA <50 copies/mL) may be used to assess virological efficacy in more detail, with viral load below the detection sensitivity,^13,14^ inflammation markers^15–18^ being evaluated as outcomes. The method of evaluating HIV-RNA with less than detection sensitivity or viral blips has the advantage of using data obtained in daily clinical practice. In Italian real-world data, about 40% of B/F/TAF users had undetectable HIV-RNA, and those with HIV-RNA less than detection sensitivity were able to maintain undetectable HIV-RNA if their estimated adherence level was about 70% or higher.^19^ Because CAB plus RPV was introduced by switching to PWH with stably suppressed HIV-RNA, in this study, the proportion of PWH with undetectable HIV-RNA at baseline was ∼80%, but this proportion was maintained over 1 year, as was treatment with oral antiretroviral drugs.^19^

ISR is an issue first introduced by CAB plus RPV LA in the history of ART and can be the major reason of switching regimens. The ISR-related discontinuation rate tended to increase with the frequency of administration, with 1% of patients in the every 8 weeks' group and 2% in the every 4 weeks' group changing their regimens because of ISR in ATLAS-2M study.^1^ Besides, there was a 60%–70% ISR at the first injection, and about 30% at the primary endpoint of 48 weeks; the number of ISRs per visit was characterized by a decrease with each visit.^2^ The ISR data in our study differed from those in the clinical trial, possibly because the ISR endpoint of pain differed depending on how the data were taken. Moreover, as in the previous clinical trials, subjective pain was less in the second injection than in the first; however, the overall percentage of respondents who reported that the pain was bothersome was higher than those in the previous phase 3 clinical trials.^1,5^

In addition, the current study suggested that the ISR differed between CAB and RPV injection sites. Pain felt at the time of injection may be more common at the site of RPV injection, while delayed myalgia-like pain may be more common at the site of CAB injection.

The pain at the time of injection is probably a physical pain associated with the administration and may be related to the viscosity of the drug, while the delayed myalgia-like pain may be due to inflammation of the muscle. Moreover, muscle inflammation can be the focus of the fever. Pyrexia has been reported as adverse events other than ISR in about 4% of patients in CAB plus RPV LA clinical trials;^1,20^ a similar frequency of pyrexia has also been reported in a phase 3 trial of CAB single agent dosing for pre-exposure prophylaxis^21^ and the ECLAIR trial, a randomized controlled trial comparing injectable CAB with saline as placebo, reported similar levels of fever and fatigue after CAB injection, but not after saline injection.^22^ Although pyrexia and fatigue in this study were less than those reported in previous clinical trials, longer-term data are required because pyrexia can develop for the first time after the third or subsequent injections. Other than ISR, there were no adverse events related to discontinuation of CAB plus RPV LA; the safety profile was favorable in Japanese PWH.

Unlike DTG, bictegravir, ritonavir, or cobicistat, CAB does not inhibit the renal organic cation transporter (OCT) 2 or the multidrug and toxin extrusion transporter (MATE) 1 and therefore is unlikely to inhibit tubular secretion of creatinine.^21,23^ The HPTN083 study used CAB alone for pre-exposure prophylaxis and showed a decrease in serum creatinine, but no data on cystatin C.^21^ In combination with RPV, neither serum creatinine nor cystatin C data have been reported to date. This study showed a decrease in cystatin C as well as serum creatinine, which may indicate improved renal function, because cystatin C is thought to be less susceptible to the tubular transporter inhibitory effects of DTG.^24^ Cystatin C is a biomarker affected by chronic inflammation, smoking, and heart disease, but none of these factors changed before or after switching to CAB plus RPV LA in this study, and multivariable analysis showed that no baseline characteristics significantly affected the absolute changes in these biomarkers.

Although CAB may decrease both serum creatinine and cystatin C, as an off-target effect rather than improving renal function, our data may represent improvement in renal function after switching to CAB plus RPV, as no studies evaluating cystatin C after discontinuation of DTG or B/F/TAF have been reported. Long-term follow-up of urinalysis is needed to determine if the renal impairment that existed before switching to CAB plus RPV LA improves, and the detailed analysis would be left for future studies.

This study has several limitations. First, it was an observational study. Due to the nature of the retrospective study, the outcomes set in this study, such as delayed pain, were based on the authors' experience, and thus the results could have been influenced by the way these endpoints were set. Second, all participants underwent OLI before administration of injectable CAB plus RPV LA. In the extended FLAIR and SOLAR studies, OLI was optional,^3,11^ but these clinical trials showed no difference in virological efficacy with or without OLI. On the other hand, OLI was mandatory before injectable CAB plus RPV in Japan during the period when this study was conducted. Finally, the Patient-Reported Outcome used in clinical trials has not been evaluated for treatment satisfaction. However, even if PWH has copayments for medical expenses in Japan and can return to their original regimen at any time in real-world medical practice, the PWH preferred to continue CAB plus RPV LA.

This is the first report on the efficacy and safety outcomes of ART with CAB plus RPV LA for PWH in Japan. There has been no literature reporting a difference in ISR between CAB and RPV injection sites or a decrease in renal function biomarkers. In conclusion, this study suggests that switching to CAB plus RPV LA may be a beneficial treatment option for Asian PWH.

## References

[1] Overton ET, Richmond G, Rizzardini G, et al. Long-acting cabotegravir and rilpivirine dosed every 2 months in adults with HIV-1 infection (ATLAS-2M), 48-week results: A randomised, multicentre, open-label, phase 3b, non-inferiority study. Lancet 2021;396(10267):1994–2005; doi: 10.1016/s0140-6736(20)32666-0 33308425

[2] Overton ET, Richmond G, Rizzardini G, et al. Long-acting cabotegravir and rilpivirine dosed every 2 months in adults with HIV-1 infection: 152-Week results from ATLAS-2M, a randomized, open-label, Phase 3b, noninferiority study. Clin Infect Dis 2023; In Press; doi: 10.1093/cid/ciad020 PMC10156123 36660819

[3] Orkin C, Bernal Morell E, Tan DHS, et al. Initiation of long-acting cabotegravir plus rilpivirine as direct-to-injection or with an oral lead-in in adults with HIV-1 infection: Week 124 results of the open-label phase 3 FLAIR study. Lancet HIV 2021;8(11):e668–e678; doi: 10.1016/s2352-3018(21)00184-3 34656207

[4] Adachi E, Ikeuchi K, Koga M, et al. Background factors in people living with HIV in Japan who switch to cabotegravir plus rilpivirine: A pilot study. J Infect Chemother 2023;29(1):109–111; doi: 10.1016/j.jiac.2022.09.008 36116718

[5] Rizzardini G, Overton ET, Orkin C, et al. Long-acting injectable cabotegravir + rilpivirine for HIV maintenance therapy: Week 48 pooled analysis of Phase 3 ATLAS and FLAIR Trials. J Acquir Immune Defic Syndr 2020;85(4):498–506; doi: 10.1097/qai.0000000000002466 33136751 PMC7592884

[6] Karver TS, Pascual-Bernaldez M, Berni A, et al. Factors associated with health care providers' preference for forgoing an oral lead-in phase when initiating long-acting injectable cabotegravir and rilpivirine in the SOLAR Clinical Trial. AIDS Patient Care STDS 2023;37(1):53–59; doi: 10.1089/apc.2022.0168 36626155

[7] Vickers AJ, Altman DG. Statistics notes: Analysing controlled trials with baseline and follow up measurements. BMJ 2001;323(7321):1123–1124; doi: 10.1136/bmj.323.7321.1123 11701584 PMC1121605

[8] Swindells S, Andrade-Villanueva JF, Richmond GJ, et al. Long-acting cabotegravir and rilpivirine for maintenance of HIV-1 suppression. N Engl J Med 2020;382(12):1112–1123; doi: 10.1056/NEJMoa1904398 32130809

[9] Orkin C, Arasteh K, Górgolas Hernández-Mora M, et al. Long-acting cabotegravir and rilpivirine after oral induction for HIV-1 infection. N Engl J Med 2020;382(12):1124–1135; doi: 10.1056/NEJMoa1909512 32130806

[10] Dandachi D, Dang BN, Lucari B, *et al.* Acceptability and preferences for long-acting antiretroviral formulations among people with HIV infection. AIDS Care 2021;33(6):801–809; doi: 10.1080/09540121.2020.1764906 32408771

[11] Moti N. Ramgopal DS-P, Alessandro Berni, et al. SOLAR 12-Month Results: Randomized Switch Trial of CAB+RPV LA VS ORAL B/FTC/TAF. 30th The Conference on Retroviruses and Opportunistic Infections (CROI) [Abstract 191]. February 19–22, 2023.

[12] Borch J, Scherzer J, Jonsson-Oldenbüttel C, et al. 6-Month Outcomes of Every 2 Months Long-Acting Cabotegravir and Rilpivirine in a Real-World Setting—Effectiveness, Adherence to Injections, and Patient Reported Outcomes of People Living With HIV in the German CARLOS Cohort. HIV Drug Therapy Glasgow [Abstract P029] Virtual and Glasgow, October 26, 2022.

[13] Underwood M, Wang R, Horton J, et al. DTG +3TC in GEMINI-1 & -2: HIV-1 Replication at <50 c/ml and VL ‘Blips' Through 144 Weeks. 11th IAS Conference on HIV Science [Abstract PEB163]. July 18–21, 2022.

[14] Santoro MM, Armenia D, Teyssou E, et al. Virological efficacy of switch to DTG plus 3TC in a retrospective observational cohort of suppressed HIV-1 patients with or without past M184V: The LAMRES Study. J Glob Antimicrob Resist 2022;31:52–62; doi: 10.1016/j.jgar.2022.07.022 35948240

[15] Adachi E, Ikeuchi K, Koga M, et al. Changes in inflammatory biomarkers when switching from three-drug regimens to dolutegravir plus lamivudine in people living with HIV. AIDS Res Hum Retroviruses 2022;38(12):881–883; doi: 10.1089/aid.2022.0115 36301933

[16] Deeks SG, Tracy R, Douek DC. Systemic effects of inflammation on health during chronic HIV infection. Immunity 2013;39(4):633–645; doi: 10.1016/j.immuni.2013.10.001 24138880 PMC4012895

[17] Hamlyn E, Stöhr W, Cooper DA, et al. The effect of short-course antiretroviral therapy initiated in primary HIV-1 infection on interleukin-6 and D-dimer levels. AIDS 2015;29(11):1355–1361; doi: 10.1097/qad.0000000000000675 25870986

[18] Kuller LH, Tracy R, Belloso W, et al. Inflammatory and coagulation biomarkers and mortality in patients with HIV infection. PLoS Med 2008;5(10):e203; doi: 10.1371/journal.pmed.0050203 18942885 PMC2570418

[19] Maggiolo FMD, Valenti DB, Teocchi RB, *et al.* Real world data on forgiveness to uncomplete adherence to Bictegravir/Emtricitabine/Tenofovir Alafenamide. J Int Assoc Provid AIDS Care 2022;21:23259582221140208; doi: 10.1177/23259582221140208 36423244 PMC9703486

[20] Orkin C, Oka S, Philibert P, et al. Long-acting cabotegravir plus rilpivirine for treatment in adults with HIV-1 infection: 96-Week results of the randomised, open-label, phase 3 FLAIR study. Lancet HIV 2021;8(4):e185–e196; doi: 10.1016/s2352-3018(20)30340-4 33794181

[21] Landovitz RJ, Donnell D, Clement ME, et al. Cabotegravir for HIV prevention in cisgender men and transgender women. N Engl J Med 2021;385(7):595–608; doi: 10.1056/NEJMoa2101016 34379922 PMC8448593

[22] Markowitz M, Frank I, Grant RM, et al. Safety and tolerability of long-acting cabotegravir injections in HIV-uninfected men (ECLAIR): A multicentre, double-blind, randomised, placebo-controlled, phase 2a trial. Lancet HIV 2017;4(8):e331–e340; doi: 10.1016/s2352-3018(17)30068-1 28546090

[23] ViiV Healthcare. Global Data Sheet for Cabotegravir (PrEP). Version 01. July 1, 2021.

[24] Yoshino Y, Koga I, Seo K, *et al.* Short communication: The clinical value of cystatin C as a marker of renal function in HIV patients receiving Dolutegravir. AIDS Res Hum Retroviruses 2017;33(11):1080–1082; doi: 10.1089/aid.2017.0074 28649847

